# A Latent Profile Analysis of Coping, Psychological Inflexibility, and Health-Related Quality of Life in Adult Regular Exercisers

**DOI:** 10.3390/sports14100452

**Published:** 2026-10-13

**Authors:** Walter Sapuppo, Antonietta Monda, Davide Giacconi, Rita Polito, Fiorenzo Moscatelli, Girolamo Di Maio, Giovanni Messina, Daniele Saccenti, Mattia Ferro, Claudia Maria Mineo, Antonietta Messina, Vincenzo Monda, Regina Gregori Grgič, Giovanni Michelini, Marco La Marra, Sergio Chieffi, Maria Giovanna Tafuri

**Affiliations:** 1 Department of Psychology, Sigmund Freud University Wien, Milan Branch, 20143 Milan, Italy; d.giacconi@phd.milano-sfu.it (D.G.); d.saccenti@milano-sfu.it (D.S.); m.ferro@milano-sfu.it (M.F.); mineo.ext@milano-sfu.it (C.M.M.); r.gregori@milano-sfu.it (R.G.G.); g.michelini@milano-sfu.it (G.M.); 2 Studi Cognitivi, Cognitive Psychotherapy School and Research Center, 20121 Milan, Italy; 3 INSPIRE Lab, Department of Psychology, Sigmund Freud University Wien, Milan Branch, 20143 Milan, Italy; 4 Department of Human Science and Quality of Life Promotion, San Raffaele Telematic University, 00166 Rome, Italy; antonietta.monda@uniroma5.it; 5 Faculty of Psychology, Sigmund Freud Privat Universität, 1020 Vienna, Austria; 6 Department of Psychology and Health Sciences, Pegaso Telematic University, 80143 Naples, Italy; rita.polito@unipegaso.it (R.P.); girolamo.dimaio@unipegaso.it (G.D.M.); 7 Department of Education and Sport Sciences, Pegaso Telematic University, 80143 Naples, Italy; 8 Department of Experimental Medicine, University of Campania “Luigi Vanvitelli”, 80138 Naples, Italy; giovanni.messina@unicampania.it (G.M.); marco.lamarra@unicampania.it (M.L.M.); sergio.chieffi@unicampania.it (S.C.); 9 Department of Precision Medicine, University of Campania “Luigi Vanvitelli”, 80138 Naples, Italy; antonietta.messina@unicampania.it; 10 Department of Economics, Law, Cybersecurity and Sports Sciences, University of Naples Parthenope, 80131 Naples, Italy; vincenzo.monda@uniparthenope.it; 11 Department of Disabilities, Fondazione Istituto Ospedaliero di Sospiro, 26048 Sospiro, Italy; 12 Department of Literary, Linguistic and Philosophical Studies, Pegaso University, 80143 Naples, Italy; mariagiovanna.tafuri@unipegaso.it

**Keywords:** latent profile analysis, coping strategies, psychological inflexibility, cognitive fusion, health-related quality of life

## Abstract

Background: Regular physical activity is generally associated with better health-related quality of life (HRQoL) and lower psychological distress, yet regular exercisers may differ in how coping strategies, psychological inflexibility, and cognitive fusion relate to perceived health and functioning. This study aimed to identify latent profiles of adult regular exercisers based on coping, psychological inflexibility, cognitive fusion, HRQoL, psychological distress, age, body mass index (BMI), and years of regular physical activity. Methods: A total of 591 regular exercisers (358 males, 60.6%; 233 females, 39.4%; mean age = 32.46 ± 13.72 years) completed the COPE-NVI-25, SF-36, GHQ-12, Acceptance and Action Questionnaire–II (AAQ-II), and Italian Cognitive Fusion Questionnaire (I-CFQ). Results: Latent profile analysis was performed using age, BMI, years of physical activity, five coping subscales, eight SF-36 domains, three GHQ-12 domains, and AAQ-II and I-CFQ scores. A three-profile solution was retained as the preferred substantive solution based on the joint consideration of statistical fit, class representation, entropy (0.87), parsimony, and interpretability. Class 1 (35.5%) showed high avoidance, poorer HRQoL, and higher distress; Class 2 (43.0%) showed near-average coping and better HRQoL; Class 3 (21.5%), comprising older, longer-term exercisers, showed the most adaptive coping and psychological flexibility profile alongside favorable HRQoL. Conclusions: These findings support person-centered approaches in sport and exercise psychology.

## 1. Introduction

Regular physical activity is generally associated with better physical and psychological health across adulthood [1,2]. Evidence from reviews suggests that physically active individuals tend to report more favorable cardiovascular, metabolic, and mental health outcomes than less active individuals; however, these associations appear to vary across populations and contexts [3]. Similar findings have been reported in sport and exercise settings, where regular activity is often associated with better health-related quality of life (HRQoL), greater well-being, and lower psychological distress [4,5]. This association, however, is not entirely consistent. Regular exercisers may differ substantially in how they manage stress, interpret internal experiences, and respond to the demands of training and everyday life, suggesting that psychological and behavioral processes may help explain variability in health-related outcomes within physically active populations [6,7]. Regular exercise, therefore, does not necessarily correspond to the same level of psychological adjustment in all individuals. Coping is among the processes most frequently examined to account for these differences [8,9,10,11]. Lazarus and Folkman [12] define coping as the cognitive and behavioral efforts used to manage demands appraised as taxing or exceeding personal resources. This framework was operationalized in the Coping Orientations to Problems Experienced inventory [13] and adapted for Italian samples as the Coping Orientations to Problems Experienced—Nuova Versione Italiana, 25 items (COPE-NVI-25), which assesses Avoidance strategies, Transcendent orientation, Positive attitude, Social support, and Problem-oriented coping [14]. In sport psychology, more adaptive forms of coping, particularly those centered on task management and active engagement, are usually associated with better adjustment, whereas avoidance and disengagement tend to be linked to poorer outcomes [15,16,17]. These coping processes are closely related to a broader construct that has received growing attention in sport and exercise research: psychological flexibility. Within the contextual behavioral literature, psychological flexibility refers to the ability to remain in contact with ongoing experience and to act consistently with personally valued goals, even when discomfort or unwanted internal events are present [18,19,20]. This construct has been described as a broad indicator of adaptive functioning, associated with more effective emotion regulation and lower vulnerability to psychological distress [21]. Its relevance may be particularly salient in exercise and sport contexts, where physical activity can involve fatigue, frustration, pain, uncertainty, and performance pressure [22,23,24]. How these experiences are interpreted and managed may influence persistence, adherence, and broader perceptions of health and functioning [25,26,27]. In the present study, psychological inflexibility and experiential avoidance were assessed with the Acceptance and Action Questionnaire–II (AAQ-II; [28]), whereas cognitive fusion was assessed with the Cognitive Fusion Questionnaire (I-CFQ; [29]). Cognitive fusion refers to the tendency to become overly entangled with one’s thoughts and to respond to them as if they accurately reflected reality [30]. These processes are relevant in physically active populations because individuals who respond rigidly to internal discomfort, avoid unwanted experiences, or overidentify with self-critical thoughts may remain vulnerable to distress despite regular exercise [31,32]. Italian versions of both instruments are available and show adequate psychometric properties [33,34]. A further set of variables concerns perceived health and general psychological distress. In the present study, HRQoL was assessed with the 36-Item Short Form Health Survey (SF-36; [35]), a widely used measure of physical, emotional, and social health status, whose Italian version has been validated and normed in large samples [36]. General distress and everyday dysfunction were measured with the 12-item General Health Questionnaire (GHQ-12), a screening instrument for non-psychotic psychiatric morbidity [37]. Its Italian version has shown satisfactory validity and test–retest reliability, together with a multidimensional structure including social dysfunction, anxiety and depression, and loss of confidence [38]. Taken together, the SF-36 and GHQ-12 provide a broad description of health-related functioning. This is particularly relevant in physically active populations, where regular exercise may coexist with psychological strain or less favorable subjective perceptions of health. Recent research suggests that these variables may combine differently across individuals, rather than being distributed along a single continuum [39,40,41]. Cluster-analytic work in sport and exercise settings has similarly shown that athletes with more adaptive coping profiles generally report more favorable health and psychological outcomes than those characterized by avoidance or disengagement [42,43]. Evidence from rugby athletes further suggests that meaningful subgroups can be identified when coping-related and health-related variables are examined jointly, supporting the use of person-centered approaches in sport and exercise research [44,45]. The present study extends this earlier work in several respects. Whereas previous research from our group examined a smaller and sport-specific sample using k-means clustering, the current study applies a model-based latent profile approach to a substantially larger and more heterogeneous sample of adult regular exercisers. It also broadens the psychological and health-related domains considered by jointly examining coping strategies, psychological inflexibility, cognitive fusion, multidimensional HRQoL, and general psychological distress. Correlational analyses remain informative, but they describe average associations between variables and do not indicate whether specific configurations of coping, psychological inflexibility, cognitive fusion, and health-related functioning are concentrated within distinct subgroups of exercisers [46,47,48]. Independent person-centered research in sport psychology has likewise identified distinct psychological and behavioral profiles among athletes, supporting the relevance of examining heterogeneity at the individual rather than exclusively at the variable level [49,50]. Latent profile analysis (LPA) is well suited to address this question because it allows individuals to be grouped according to shared multivariate response patterns [51]. LPA is a model-based person-centered technique used to identify unobserved subgroups of individuals who show similar patterns across a set of continuous indicators [51,52]. In the present study, age, BMI, and years of regular physical activity were included as profile indicators rather than external covariates because the analytic aim was to identify multivariate configurations jointly reflecting demographic, behavioral, psychological, and health-related characteristics. Accordingly, these variables were treated as components of the latent profiles themselves rather than as independent predictors or correlates of profile membership. In our dataset, coping, psychological inflexibility, cognitive fusion, HRQoL, and general distress were meaningfully interrelated: avoidance, inflexibility, and fusion showed the least favorable pattern of associations, whereas positive attitude and problem orientation showed the opposite trend. This correlational structure does not, by itself, imply the presence of discrete groups. It does, however, provide a rationale for examining whether these variables organize into distinct profiles within a heterogeneous sample of adult regular exercisers [53,54]. The aim of the present study was therefore to identify latent profiles of adult regular exercisers based on coping strategies, psychological inflexibility, cognitive fusion, HRQoL, general psychological distress, age, BMI, and years of regular physical activity. A further aim was to characterize the identified profiles from a health psychology perspective, with particular attention to whether more adaptive configurations of coping and psychological flexibility tended to co-occur with more favorable perceptions of health and lower distress.

## 2. Materials and Methods

### 2.1. Participants

Participants were adult regular exercisers recruited from the general population and from amateur, semi-professional, and professional sports clubs. Recruitment took place on a voluntary basis through online platforms, including social media and email, as well as through direct contact with sports organizations, gyms, and clubs. Individuals were eligible if they were 18 years of age or older and were currently engaged or had previously been engaged in regular sport or exercise at either competitive or amateur level. In line with previous work from the same research group, inclusion criteria required at least one year of regular training experience. For the purposes of the study, regular physical activity was operationally defined as structured and planned physical activity practiced continuously for at least one year, irrespective of competitive level or discipline. Because weekly frequency and current training volume were not systematically recorded, this definition captures the duration and continuity of engagement rather than a fixed frequency threshold; this limitation is further addressed in Section Limitations. The final sample comprised 591 adults.

### 2.2. Study Design and Procedure

This was a non-clinical, cross-sectional study. Following ethical approval on 30 July 2024, participant recruitment and data collection were conducted through March 2025 using an online survey. The full questionnaire battery was administered via Google Forms. Before accessing the survey, potential participants received an information sheet describing the aims of the study, the voluntary nature of participation, and the confidential handling and analysis of the data. They were then required to provide informed consent for the processing of their data for scientific and research purposes before proceeding to the questionnaires. No monetary or material incentives were offered. No personally identifying information was collected, and responses were stored in anonymized form on secure university servers, in accordance with institutional guidelines. The study, including all procedures for data collection and storage, was approved by the Ethics Committee of Sigmund Freud University, Ethics Commission of the Faculty of Psychotherapy Science and the Faculty of Psychology (approval reference BD5VKJDAC4UJIC91006; 30 July 2024). All procedures were carried out in accordance with the Declaration of Helsinki.

### 2.3. Measures

Health-related quality of life (HRQoL) was assessed using the Italian version of the 36-Item Short Form Health Survey (SF-36). The SF-36 is a self-report questionnaire comprising 36 items grouped into eight domains: Physical Functioning (PF), Role Physical (RP), Role Emotional (RE), Vitality (VT), Mental Health (MH), Social Functioning (SF), Bodily Pain (BP), and General Health (GH). Higher scores indicate better perceived health status and quality of life [36]. In the present sample, internal consistency varied across domains (α ranging from 0.40 to 0.88; see Table 1). General psychological distress and everyday dysfunction were assessed using the 12-item General Health Questionnaire (GHQ-12). The GHQ-12 is a brief self-report screening instrument designed to detect non-psychotic psychiatric morbidity in community and primary care samples. In this study, a three-factor scoring model including Social Dysfunction, Anxiety and Depression, and Loss of Confidence was used, together with a total score [38]. Higher scores reflect greater levels of distress and impairment. Internal consistency in the present sample was satisfactory (α = 0.72 for the total score, and α ranging from 0.73 to 0.74 for the subscales). Coping strategies were assessed with the Coping Orientations to Problems Experienced—Nuova Versione Italiana, 25 items (COPE-NVI-25). The COPE-NVI-25 is a 25-item self-report questionnaire that evaluates five dimensions: Avoidance strategies, Transcendent orientation, Positive attitude, Social support, and Problem-oriented coping. Items are rated on a four-point scale from 1 (“Not at all”) to 4 (“Very much”), and subscale scores are computed by summing the relevant items, with higher scores indicating a greater tendency to use that strategy [14]. In the present sample, internal consistency for the five scales ranged from 0.68 (Avoidance strategies) to 0.96 (Transcendent orientation). Psychological inflexibility and experiential avoidance were measured using the Acceptance and Action Questionnaire–II (AAQ-II). The AAQ-II is a self-report instrument assessing the tendency to respond rigidly to unwanted internal experiences and to avoid them even when doing so conflicts with personally valued goals. It consists of seven items rated on a Likert-type scale, with higher total scores indicating higher levels of psychological inflexibility [28].

In this study, the validated Italian version of the AAQ-II was used, which has shown satisfactory dimensionality, internal consistency, and convergent validity [33]. Internal consistency in the present sample was good (α = 0.87). Cognitive fusion was assessed with the Italian version of the Cognitive Fusion Questionnaire (I-CFQ). The I-CFQ is a self-report measure that evaluates the extent to which individuals become entangled with their thoughts and respond to them as literal truths. The questionnaire comprises seven items, rated on a Likert-type scale, with higher scores reflecting higher levels of cognitive fusion. The Italian I-CFQ has shown a unidimensional structure and good reliability [34]. In the present sample, internal consistency was excellent (α = 0.93). Demographic and physical-activity-related information was collected through a brief ad hoc questionnaire. Participants reported their age, gender, nationality, height, and weight; height and weight were used to compute BMI (kg/m^2^). Additional items assessed years of regular physical activity, perceived regularity of physical activity over time, and the main motivation for engaging in physical activity.

### 2.4. Statistical Analysis

Descriptive statistics and frequencies were computed for demographic and continuous variables extracted from the self-report questionnaires, while Cronbach’s α was calculated to assess the internal consistency of the adopted measures. No missing data were present for the variables included in the descriptive, correlation, and latent profile analyses; therefore, all analyses were conducted on the full sample of 591 participants. The number of individuals who accessed the survey link without completing it was not recorded; consequently, participation and response rates could not be computed, and the analytic sample reflects only fully completed and consented questionnaires. Subsequently, bivariate Pearson correlation analyses were performed to examine the relationships among general health, health-related quality of life, coping strategies, psychological inflexibility, and cognitive fusion in the whole sample. Latent profile analysis (LPA) was then used to identify profiles (or subtypes within the dataset) characterized by different combinations of demographic characteristics and levels of general health, health-related quality of life, coping strategies, psychological inflexibility, and cognitive fusion. The LPA was conducted using the ‘tidyLPA’ package [55]. Solutions containing one to five profiles were tested, employing the default model parameters (i.e., Model 1, with equal variances and covariances fixed to zero). To assess the robustness of the profile solution to alternative model specifications, two additional models were also examined: Model 2, allowing variances to vary across profiles while constraining covariances to zero, and Model 3, assuming equal variances and covariances across profiles. An additional specification allowing both variances and covariances to vary freely across profiles (Model 6) was also considered but could not be estimated within the Mclust framework implemented in tidyLPA. The variables included in the models were participants’ age, body mass index, years of regular physical activity, the five COPE-NVI-25 subscale scores, the eight SF-36 subscale scores, the three GHQ-12 subscale scores, as well as the AAQ-II and I-CFQ total scores. All values were converted into z-scores. To inform the selection of the preferred profile solution, six commonly used fit indices were considered, namely the Akaike Information Criterion (AIC), Bayesian Information Criterion (BIC), Consistent Akaike Information Criterion (CAIC), Kullback Information Criterion (KIC), Classification Likelihood Criterion (CLC), and Appropriate Weight of Evidence Criterion (AWE). For all indices, lower scores indicated a better-fitting model. Additionally, entropy was assessed for each solution, with scores ranging from 0 to 1; higher values indicated greater classification certainty, and values above 0.80 were considered acceptable [56]. Consistent with recommendations in the literature [52], the presence of profiles comprising fewer than 5% of participants was considered as one practical criterion in model selection, alongside statistical fit and interpretability. Two additional sensitivity analyses were conducted to examine the dependence of the retained profile configuration on indicator selection. First, the LPA was repeated after excluding age, BMI, and years of regular physical activity. Second, the analysis was repeated after excluding the SF-36 Social Functioning domain, which showed low internal consistency in the present sample (Cronbach’s α = 0.40). For both sensitivity analyses, one- to five-profile solutions were examined using the same primary model specification and model-selection criteria. The significance threshold was set at *p* < 0.05 (two-tailed). All statistical analyses were conducted in R using appropriate packages and custom scripts (Version 4.5.3; https://www.r-project.org/, accessed on 1 April 2026).

## 3. Results

### 3.1. Descriptive Statistics

Of the 591 participants, 358 (60.6%) were male and 233 (39.4%) were female. The vast majority of participants were Italian (98.5%), while the remaining participants were French (0.3%), Swiss (0.3%), British (0.2%), Indian (0.2%), Peruvian (0.2%), Russian (0.2%), and Slovenian (0.2%). The mean age of the sample was 32.46 ± 13.72 years, while the mean body mass index (BMI) was 24.18 ± 4.05 kg/m^2^. Participants reported engaging in regular physical activity for a mean duration of 16.61 ± 11.91 years. However, participants reported different patterns of physical activity consistency over time, with 61.8% of the participants stating that their level of physical activity had remained generally stable across the years, 30.1% reporting having been more physically active in the past, and 8.1% indicating that they currently engage in more physical activity than they did previously. Motivations for engaging in regular physical activity were also heterogeneous, including improvement of physical and psychological health (62.9%), improvement of body image (10.7%), entertainment and social interaction (10.0%), improvement of athletic performance (8.3%), and competition (8.1%). Descriptive statistics for continuous variables related to general health, quality of life, coping strategies, psychological inflexibility, and cognitive fusion are presented in Table 1.

### 3.2. Pearson Correlations

**Figure 1:**
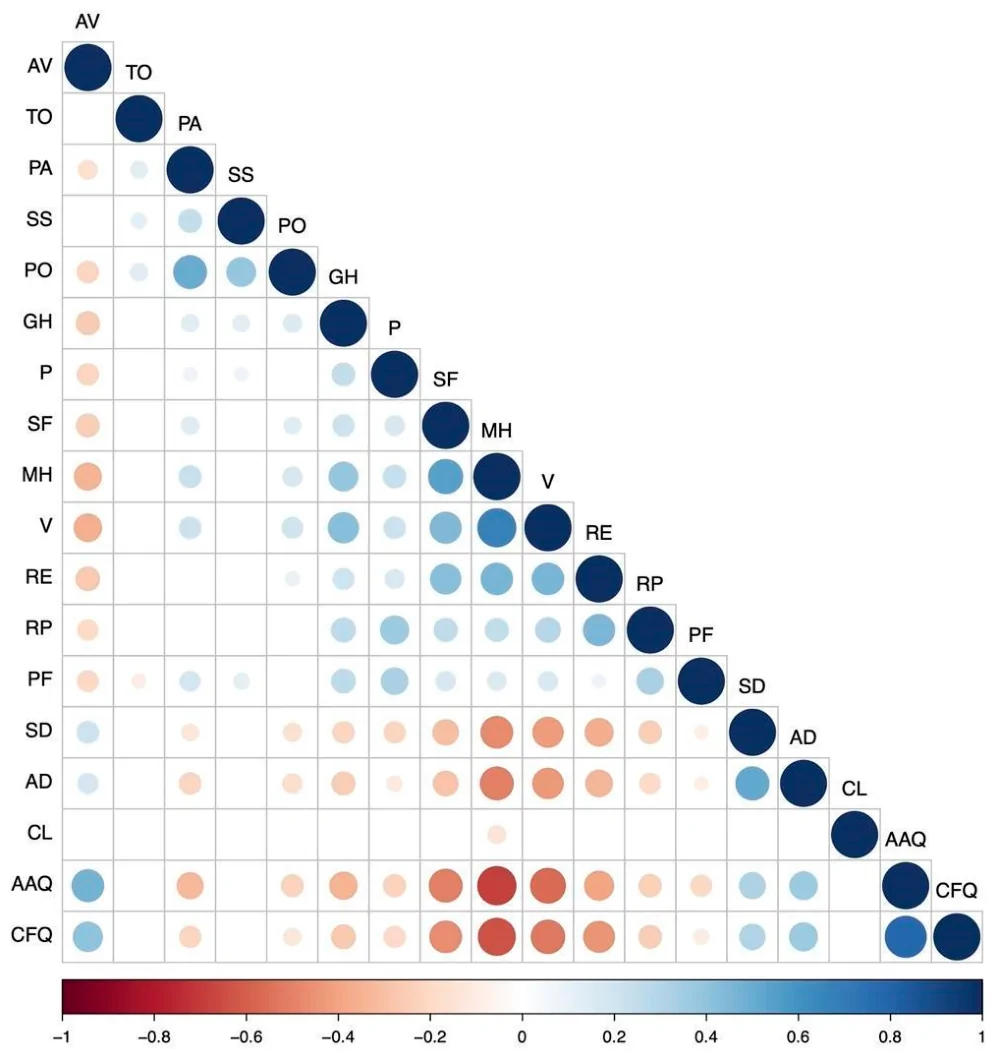
Corrplot of the relationships among self-report measures. Only statistically significant correlations were retained, whereas non-significant associations were dropped. Positive correlations are displayed in blue and negative correlations in red, with larger dots and greater color intensity indicating higher absolute Pearson correlation coefficients. Overall, maladaptive psychological processes, such as avoidance, psychological inflexibility, and cognitive fusion, were associated with poorer health-related quality of life and worse general health, whereas adaptive coping strategies, including positive attitude and problem orientation, showed the opposite pattern of associations. In particular, psychological inflexibility and cognitive fusion exhibited the strongest negative correlations with mental health, social functioning, and vitality. AV, Avoidance; TO, Transcendent orientation; PA, Positive attitude; SS, Social Support; PO, Problem Orientation; GH, General Health; P, Bodily Pain; SF, Social Functioning; MH, Mental Health; V, Vitality; RE, Role Limitation—Emotional; RP, Role Limitation—Physical; PF, Physical Functioning; SD, Social Dysfunction; AD, Anxiety and Depression; CL, Loss of Confidence; AAQ, Acceptance and Action Questionnaire–II Total Score; CFQ, Italian Cognitive Fusion Questionnaire Total Score.” These labels refer to the AAQ-II and I-CFQ, respectively.

Significant yet weak negative correlations were found between avoidance and all SF-36 domains (*r*(589) ranging from −0.20 to −0.34). Positive correlations emerged between avoidance and the social dysfunction (*r*(589) = 0.22) and anxiety and depression (*r*(589) = 0.19) domains of the GHQ-12, as well as AAQ-II (r(589) = 0.48) and I-CFQ (r(589) = 0.40) total scores. Transcendent orientation was negatively, albeit very weakly, associated with physical functioning (*r*(589) = −0.09). Positive attitude was positively correlated with the majority of SF-36 domains (*r*(589) ranging from 0.09 to 0.22). Conversely, negative correlations were observed between positive attitude and the social dysfunction (*r*(589) = −0.14) and anxiety and depression (*r*(589) = −0.22) domains of the GHQ-12, as well as with AAQ-II (r(589) = −0.32) and I-CFQ (r(589) = −0.21) total scores. Social support showed weak positive correlations with the general health (*r*(589) = 0.13), bodily pain (*r*(589) = 0.09), and physical functioning (*r*(589) = 0.11) domains of the SF-36. Problem orientation was positively associated with most SF-36 domains (*r*(589) ranging from 0.10 to 0.20). In contrast, negative associations were found between problem orientation and the social dysfunction (*r*(589) = −0.16) and anxiety and depression (*r*(589) = −0.18) domains of the GHQ-12, as well as AAQ-II (*r*(589) = −0.22) and I-CFQ (*r*(589) = −0.14) total scores. The GHQ-12 Social Dysfunction (r(589) ranging from −0.09 to −0.47) and Anxiety and Depression (r(589) ranging from −0.09 to −0.51) domains were negatively correlated with all SF-36 domains. Loss of confidence was negatively associated with mental health (*r*(589) = −0.14). Psychological inflexibility (AAQ-II total score) was negatively associated with all SF-36 domains (*r*(589) ranging from −0.20 to −0.68) and positively correlated with the social dysfunction (*r*(589) = 0.32) and anxiety and depression (*r*(589) = 0.36) domains of the GHQ-12. The strongest, although still moderate, negative associations were observed for mental health (*r*(589) = −0.68), social functioning (*r*(589) = −0.50), and vitality (*r*(589) = −0.56). Cognitive fusion (I-CFQ total score) showed a pattern similar to that of AAQ-II, with a strong positive correlation between the two measures (*r*(589) = 0.79). I-CFQ total scores were negatively associated with all SF-36 domains (*r*(589) ranging from −0.11 to −0.64) and positively associated with both the social dysfunction (*r*(589) = 0.30) and anxiety and depression (*r*(589) = 0.37) domains of the GHQ-12. The strongest, although still moderate, negative associations were found for mental health (*r*(589) = −0.64), social functioning (*r*(589) = −0.47), and vitality (*r*(589) = −0.52). See Figure 1 for a graphical representation.

### 3.3. Latent Profile Analysis

Information-criterion values were estimated for Models 1, 2, and 3, each employing a different variance and covariance specification (see Table 2).

Notably, Model 2 could not be estimated beyond the one-class solution, indicating that allowing class-specific variances while constraining covariances to zero resulted in non-estimable solutions in the present sample. Regarding Model 3, the three-, four-, and five-profile solutions showed better information-criterion values than the two-class solution. Nevertheless, such solutions included one or more very small profiles (<5% of the sample, N < 30) (see Table 3). Similarly, in Model 1, the four- and five-profile solutions yielded better information-criterion values than the three-profile solution. However, both solutions included a small, non-representative profile comprising less than 5% of the sample (N < 30) (see Table 3). Therefore, considering statistical fit alongside class size, parsimony, and substantive interpretability, the three-profile solution of Model 1 was retained as the preferred substantive solution.

**Figure 2:**
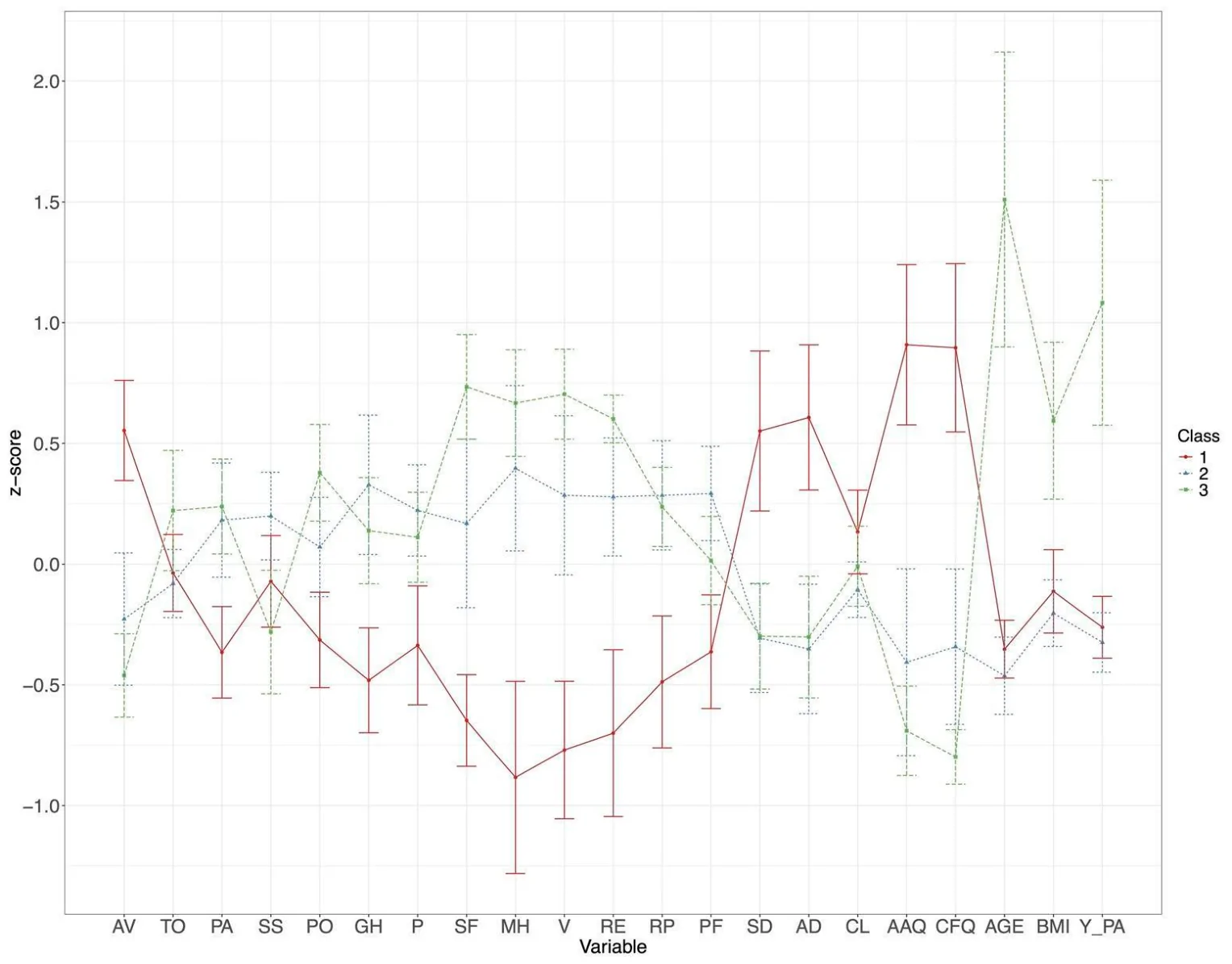
Standardized z-scores ± standard errors are displayed for all variables used for classification. Overall, the three classes showed distinct psychological and health-related profiles. Class 1 (in red) was characterized by maladaptive coping patterns, poorer health-related quality of life, higher psychological distress, and greater psychological inflexibility and cognitive fusion. Class 2 (in blue) displayed an intermediate and generally adaptive profile, with average coping strategies, better perceived quality of life, and lower psychological distress. Finally, Class 3 (in green) was characterized by the most adaptive psychological profile, including high positive attitude and problem orientation, low avoidance, more favorable mental and social health outcomes and greater psychological flexibility with lower cognitive fusion. COPE-NVI-25, 25-Item Coping Orientation to Problems Experienced–New Italian version; GHQ-12, 12-Item General Health Questionnaire; A/F, Acceptance/Fusion; DEMO, Demographics; AV, Avoidance; TO, Transcendent orientation; PA, Positive attitude; SS, Social Support; PO, Problem Orientation; GH, General Health; P, Bodily Pain; SF, Social Functioning; MH, Mental Health; V, Vitality; RE, Role Limitation—Emotional; RP, Role Limitation—Physical; PF, Physical Functioning; SD, Social Dysfunction; AD, Anxiety and Depression; CL, Loss of Confidence; AAQ-II, Acceptance and Action Questionnaire–II Total Score; I-CFQ, Italian Cognitive Fusion Questionnaire Total Score; BMI, Body Mass Index; Y_PA, Years Practicing Regular Physical Activity.

The classes within the retained three-profile solution differed in terms of demographic characteristics, levels of general health, health-related quality of life, coping strategies, psychological inflexibility, and cognitive fusion (see Figure 2).

Class 1 comprised 210 participants (35.53%), with a mean age of 27.60 ± 9.74 and a mean BMI of 23.72 ± 4.12. Of these participants, 104 were male (49.52%), whereas 106 were female (50.48%). They reported engaging in regular physical activity for a mean of 13.53 ± 8.48 years. Regarding coping strategies, this class was characterized by high avoidance, low positive attitude, low problem orientation, average transcendent orientation, and average social support. Turning to health-related quality of life, this class showed low scores in all SF-36 domains. Accordingly, these participants reported high scores on the Social Dysfunction and Anxiety and Depression domains of the GHQ-12, while loss of confidence scores were close to the average. High scores were also observed for the AAQ-II and I-CFQ, indicating higher levels of psychological inflexibility and cognitive fusion.

Class 2 comprised 254 participants (42.98%), with a mean age of 26.03 ± 6.35 and a mean BMI of 23.37 ± 3.29. Of these participants, 150 were male (59.06%), whereas 104 were female (40.94%). They reported practicing regular physical activity for a mean of 12.70 ± 7.26 years. Although demographic characteristics were similar to those of Class 1, participants in Class 2 showed scores close to the average in all COPE-NVI-25 domains. Moreover, these participants reported higher scores in all SF-36 domains compared to those belonging to Class 1. Likewise, participants classified in Class 2 showed low scores on the Social Dysfunction and Anxiety and Depression domains of the GHQ-12, while loss of confidence scores remained close to the average. These participants also reported lower total scores on both the AAQ-II and I-CFQ, indicating greater psychological flexibility and lower cognitive fusion.

Class 3 comprised 127 participants (21.49%), with a mean age of 53.35 ± 8.99 and a mean BMI of 26.56 ± 4.43. Of these participants, 104 were male (81.89%), whereas 23 were female (18.11%). They reported engaging in regular physical activity for a mean of 29.53 ± 14.96 years. In addition to showing demographic characteristics that differed from those of the other classes, Class 3 was characterized by low avoidance and low social support, along with high transcendent orientation, high positive attitude, and high problem orientation. Regarding health-related quality of life, these participants reported higher scores in the following SF-36 domains: social functioning, mental health, vitality, role limitations due to emotional concerns, and role limitations due to physical conditions. Conversely, general health, bodily pain, and physical functioning scores were close to the average. Furthermore, participants in Class 3 showed low scores on the Social Dysfunction and Anxiety and Depression domains of the GHQ-12, while loss of confidence scores remained close to the average. These participants also displayed lower total scores on both the AAQ-II and I-CFQ compared with Classes 1 and 2, thus suggesting greater psychological flexibility and lower cognitive fusion. See Table 4 for a summary of the characteristics of the three profiles.

### 3.4. Sensitivity Analyses

When age, BMI, and years of regular physical activity were excluded from the indicator set, class proportions changed substantially relative to the primary model (Class 1: N = 90, 15.23%; Class 2: N = 286, 48.39%; Class 3: N = 215, 36.38%; entropy = 0.868), indicating that these demographic and activity history indicators contribute to class composition. However, inspection of the standardized profile plots indicated that the underlying psychological and health-related configuration remained recognizable across classes, suggesting that class differentiation was not attributable solely to these indicators. As in the primary analysis, information criteria favored more complex solutions; the four-profile solution included a class close to the 5% threshold (5.08%), while the five-profile solution contained two classes below 5% (3.72% and 4.23%). Detailed fit indices are provided in Supplementary Table S1 and standardized profiles in Supplementary Figure S1. When the SF-36 Social Functioning indicator was excluded, given its low internal consistency (α = 0.40), class proportions remained closely comparable to the primary model (Class 1: N = 202, 34.18%; Class 2: N = 260, 43.99%; Class 3: N = 129, 21.83%; entropy = 0.886), indicating that the core three-profile configuration was preserved after removal of this low-reliability indicator. Unlike the other sensitivity analyses, the four- and five-profile solutions in this analysis did not contain classes below 5% (minimum class sizes of 8.12% and 5.25%, respectively), and information criteria continued to improve up to the five-profile solution. Inspection of the standardized profiles indicated that the additional classes in the four- and five-profile solutions mainly represented further subdivisions of the broader configurations identified by the three-profile solution rather than clearly distinct substantive patterns. The three-profile solution was therefore retained as the most parsimonious representation for this sensitivity analysis. Detailed fit indices are provided in Supplementary Table S2 and standardized profiles in Supplementary Figure S2.

## 4. Discussion

The present study aimed to identify psychological and health-related latent profiles in adult regular exercisers by jointly considering coping strategies, psychological inflexibility, cognitive fusion, HRQoL, general psychological distress, and selected demographic indicators. Because age, BMI, and years of regular physical activity were included as indicators in the LPA, these characteristics contributed directly to the definition of the latent profiles and should not be interpreted as independent correlates of class membership. Importantly, a sensitivity analysis excluding age, BMI, and years of regular physical activity reproduced a broadly comparable three-profile psychological and health-related configuration, suggesting that the primary differentiation among profiles was not attributable solely to these demographic and activity history indicators. The results indicate meaningful heterogeneity within this population, as the three-profile solution differentiated subgroups with distinct configurations of coping, psychological flexibility-related processes, perceived health, and distress. A three-profile solution was retained as the preferred substantive solution, identifying a more vulnerable profile, an intermediate profile, and a more adaptive profile. The first point deserves emphasis. Regular physical activity, by itself, did not correspond to a uniformly positive psychological configuration. Although all participants were regular exercisers, one class was characterized by elevated avoidance, higher psychological inflexibility, higher cognitive fusion, poorer HRQoL, and more marked distress symptoms. This finding is consistent with evidence that maladaptive psychological features, including body-image and eating-related difficulties, may occur among individuals engaged in regular exercise [57]. The correlational analyses showed a convergent pattern. Avoidance was negatively associated with all HRQoL domains and positively associated with psychological distress, inflexibility, and cognitive fusion. By contrast, Positive Attitude and Problem Orientation showed a more favorable pattern, with modest but coherent positive associations with HRQoL and negative associations with distress-related indices. AAQ-II and I-CFQ scores were strongly interrelated and showed the strongest negative correlations with Mental Health, Social Functioning, and Vitality. Taken together, these findings indicate that psychological inflexibility and cognitive fusion co-occurred with less favorable mental and social functioning within the multivariate configurations observed in this sample. The first latent profile, comprising about one-third of the sample, appears to represent a psychologically vulnerable subgroup. Its combination of high avoidance, low positive attitude, low problem orientation, elevated inflexibility and cognitive fusion, and poorer HRQoL across domains points to a maladaptive configuration that cannot be reduced to a single variable. This multivariate interpretation is consistent with person-centered research in sport showing that psychologically relevant characteristics and interpersonal experiences may cluster into distinct profiles [58,59]. The second profile, which was the largest, appeared more balanced. Participants in this group were relatively similar to those in Class 1 in age, BMI, and years of physical activity, yet they reported lower distress, greater psychological flexibility, and better HRQoL. This contrast indicates that different psychological and health-related configurations may be observed even among individuals with comparable demographic and activity-related characteristics [60]. The third profile showed the most adaptive pattern within the present sample. Participants in this class were older, had practiced regular physical activity for much longer, and showed low avoidance, high positive attitude, high problem orientation, lower inflexibility and cognitive fusion, and more favorable scores in several mental and social HRQoL domains. This pattern is compatible with the possibility that a longer history of regular physical activity is associated with a more adaptive psychological configuration, although the cross-sectional design does not allow causal inference. An alternative, equally plausible interpretation is that psychological flexibility and adaptive self-regulatory strategies may contribute to the maintenance of physical activity over time [61,62]. Another relevant aspect concerns social support. In the correlational analyses, social support showed weak positive associations with selected health domains, yet the most adaptive latent profile within the present sample was characterized by relatively low social support despite favorable functioning overall. This pattern suggests that the role of social support may not be captured by a simple linear positive association and may depend on how it combines with other coping strategies and psychological flexibility-related processes. One possible hypothesis is that, in older or more experienced exercisers, lower reliance on social support may reflect greater autonomy rather than poorer resources; however, autonomy was not assessed in the present study, and this interpretation should therefore be examined in future research. The present findings are also consistent with the usefulness of person-centered approaches in sport and exercise psychology [63,64,65,66]. Variable-centered analyses describe average associations, whereas the present LPA suggests that coping, inflexibility, fusion, distress, and HRQoL may cluster in specific ways across subgroups. This has applied relevance because screening approaches based only on mean levels may overlook subgroups characterized by less favorable multivariate configurations despite sustained involvement in exercise. Future intervention research could examine whether psychological flexibility and coping represent useful targets for promoting well-being among regularly active individuals. Future studies could examine whether individuals showing characteristics similar to the first profile benefit from interventions targeting avoidance, cognitive fusion, and adaptive coping responses, including positive attitude and problem-oriented coping. More broadly, this intervention-oriented implication is consistent with sport psychology research applying cognitive-behavioral and rational-emotive approaches to athletes’ stress appraisals, emotions, beliefs, and performance-related outcomes [67,68,69,70,71,72].

### Limitations

Several limitations should be acknowledged. First, the cross-sectional nature of the design precludes causal inference. The data do not clarify whether coping and flexibility shaped the identified profiles over time or whether longer and more stable engagement in physical activity contributed to these psychological characteristics. Second, all measures were self-reported. This introduces the possibility of recall bias, shared method variance, and socially desirable responding, particularly for anthropometric variables and psychological constructs. Third, although the sample was large and heterogeneous in age and activity history, the available dataset did not allow a more detailed stratification by sport type, training load, or competitive status within the LPA model. These factors may meaningfully shape both coping and perceived health. Accordingly, some of the between-profile heterogeneity observed in the present study may also reflect differences in sport discipline, competitive level, training characteristics, or participation in individual versus team sports. Because these characteristics were not incorporated into the present profile model, their specific contribution to the identified configurations cannot be disentangled from the psychological and health-related differences described here. Fourth, the construct labeled here as psychological inflexibility was assessed through the AAQ-II, while cognitive fusion was assessed separately through the I-CFQ. Although both are theoretically relevant, they capture partially overlapping processes, and their joint inclusion may reflect a broader distress-related dimension in addition to distinct contextual behavioral mechanisms.

A further limitation concerns the internal consistency of selected subscales. Although reliability was adequate for most measures, a markedly low Cronbach’s α (0.40) was observed for the SF-36 Social Functioning domain, indicating poor internal consistency and substantial measurement error; findings involving this domain should therefore be interpreted with particular caution and regarded as exploratory. However, a sensitivity analysis excluding the Social Functioning indicator reproduced the core three-profile configuration, indicating that the substantive pattern identified in the primary analysis was preserved after removal of this indicator. A comparatively lower value was also observed for General Health (α = 0.59) and, to a lesser extent, for the COPE-NVI-25 Avoidance scale (α = 0.68). These values indicate greater measurement error for these specific indicators and may have reduced the precision with which the corresponding constructs contributed to the latent profile solution. This possibility is also consistent with the strong association observed between AAQ-II and I-CFQ scores in the present sample (r = 0.79), together with their associations with several distress- and HRQoL-related domains. Accordingly, some of the separation among the identified profiles may reflect a broader shared dimension of psychological distress or maladaptive functioning, rather than fully independent contributions of each construct. The profile labels and their substantive interpretation should therefore be considered with appropriate caution. A further methodological limitation concerns the specification of the latent profile model. The present analysis was conducted using the default tidyLPA parameterization, assuming equal variances across profiles and covariances fixed to zero. Sensitivity analyses were conducted using alternative variance–covariance specifications. Model 2 could not be estimated beyond the one-profile solution, whereas Model 3 yielded better values on several information criteria but generated a poorly represented class comprising only 3.21% of the sample in its three-profile solution, with comparably small classes in its four- and five-profile solutions. Thus, no alternative specification yielded a clearly preferable solution combining statistical fit, adequate class representation, and substantive interpretability. Model 1 was therefore retained as the primary specification. Nevertheless, these differences indicate that the precise latent class structure remains partly dependent on model specification and should be replicated in independent samples. A related limitation concerns the operational definition of regular physical activity, which was based on duration of engagement (at least one year) and self-reported regularity over time, without a standardized quantitative threshold for weekly frequency or current training volume. Future studies should incorporate such metrics to allow a more precise characterization of exercise regularity. Similarly, because the number of individuals who accessed but did not complete the survey was not recorded, response and completion rates could not be estimated, and self-selection among respondents cannot be excluded. Finally, the profiles identified in this study should not be interpreted as fixed categories. They are best understood as probabilistic groupings derived from the observed data and may vary across samples and contexts.

## 5. Conclusions

The present findings indicate that adult regular exercisers are not psychologically homogeneous. Coping strategies, psychological inflexibility, cognitive fusion, distress, and HRQoL were represented by three latent profiles, ranging from a more maladaptive and vulnerable configuration to a more adaptive pattern characterized by greater flexibility and better health-related functioning. Regular physical activity may therefore coexist with substantial psychological variability. This pattern may have practical relevance. One subgroup of exercisers showed poorer HRQoL, higher distress, greater avoidance, and higher levels of psychological inflexibility and cognitive fusion despite sustained engagement in physical activity. Another subgroup showed a more adaptive profile, marked by higher positive attitude, stronger problem orientation, lower avoidance, and better mental and social functioning. This heterogeneity suggests that mental health screening in active populations should not rely solely on the presence of regular exercise behavior. Overall, the data are consistent with an association between coping style, psychological flexibility, and well-being in regular exercisers. These findings also suggest that future intervention studies could further examine whether interventions designed to strengthen psychological flexibility can improve well-being in physically active populations [73].

## Figures and Tables

**Table 1:** Descriptive statistics and reliability coefficients for study measures.

Variable	Mean	SD	Range	α
SF-36				
Role Physical	81.09	29.67	0.00–100.00	0.76
Role Emotional	69.49	38.22	0.00–100.00	0.78
Social Functioning	64.28	24.56	0.00–100.00	0.40
Physical Functioning	94.59	10.29	25.00–100.00	0.82
Bodily Pain	82.80	19.56	10.00–100.00	0.79
General Health	66.58	14.96	20.00–100.00	0.59
Vitality	55.66	15.48	5.00–100.00	0.71
Mental Health	63.97	16.62	4.00–100.00	0.82
GHQ-12				
Total Score	16.61	4.59	3.00–32.00	0.72
Social Dysfunction	8.47	2.65	0.00–17.00	0.74
Anxiety and Depression	5.75	2.44	0.00–12.00	0.73
Loss of Confidence	2.39	1.03	0.00–6.00	0.74
COPE-NVI-25				
Avoidance Strategies	11.35	4.06	5.00–29.00	0.68
Transcendent Orientation	6.43	4.52	4.00–24.00	0.96
Positive Attitude	25.74	5.95	6.00–36.00	0.87
Social Support	18.73	6.22	5.00–30.00	0.91
Problem Orientation	19.61	4.63	5.00–30.00	0.80
AAQ-II				
Total Score	30.62	10.33	10.00–67.00	0.87
I-CFQ				
Total Score	20.85	9.43	7.00–49.00	0.93

Abbreviations: SD, Standard Deviation; α, Cronbach’s α; SF-36, 36-Item Short Form Survey; COPE-NVI-25, 25-Item Coping Orientation to Problems Experienced–New Italian version; GHQ-12, 12-Item General Health Questionnaire; AAQ-II, Acceptance and Action Questionnaire–II; I-CFQ, Italian Cognitive Fusion Questionnaire.

**Table 2:** Fit index values for the latent profile analysis solutions.

Model	Classes	AIC	AWE	BIC	CAIC	CLC	KIC	Entropy
1 ^†^	1	88,255.04	88,831.11	88,439.07	88,481.07	88,173.04	88,300.04	1.000000
	2	86,533.45	87,412.57	86,813.89	86,877.89	86,407.20	86,600.45	0.875938
	**3**	**86,102.82**	**87,284.75**	**86,479.65**	**86,565.65**	**85,932.56**	**86,191.82**	**0.869419**
	4	85,682.23	87,166.93	86,155.46	86,263.46	85,467.99	85,793.23	0.884741
	5	85,482.00	87,269.47	86,051.64	86,181.64	85,223.80	85,615.00	0.901683
2 ^††^	1	88,254.54	88,830.61	88,438.57	88,480.57	88,172.54	88,299.54	1.000000
	2	–	–	–	–	–	–	–
	3	–	–	–	–	–	–	–
	4	–	–	–	–	–	–	–
	5	–	–	–	–	–	–	–
3 ^‡^	1	84,582.19	88,048.62	85,686.40	85,938.40	84,080.19	84,837.19	1.000000
	2	84,590.43	88,360.39	85,791.04	86,065.04	84,043.69	84,867.43	0.633712
	3	83,948.50	88,020.62	85,245.52	85,541.52	83,358.42	84,247.50	0.960432
	4	83,948.52	88,323.62	85,341.94	85,659.94	83,314.26	84,269.52	0.869668
	5	83,736.30	88,414.28	85,226.12	85,566.12	83,057.95	84,079.30	0.826547

Note: ^†^ = equal variances and zero covariances; ^††^ = varying variances and zero covariances; ^‡^ = equal variances and equal covariances. Abbreviations: AIC, Akaike Information Criterion; AWE, Appropriate Weight of Evidence Criterion; BIC, Bayesian Information Criterion; CAIC, Consistent Akaike Information Criterion; CLC, Classification Likelihood Criterion; KIC, Kullback Information Criterion. Bold values indicate the retained three-profile solution of Model 1.

**Table 3:** Class sizes and proportions across latent profile solutions for Model 1 and Model 3.

Model	N of Classes	Class	N	%
1 ^†^	1	1	591	100.00
	2	1	264	44.67
		2	327	55.33
	3	1	210	35.53
		2	254	42.98
		3	127	21.49
	4	1	25	4.23 *
		2	199	33.67
		3	245	41.46
		4	122	20.64
	5	1	80	13.54
		2	242	40.95
		3	129	21.83
		4	111	18.78
		5	29	4.91 *
3 ^‡^	1	1	591	100.00
	2	1	415	70.22
		2	176	29.78
	3	1	19	3.21 *
		2	426	72.08
		3	146	24.71
	4	1	19	3.21 *
		2	82	13.87
		3	348	58.88
		4	142	24.04
	5	1	18	3.05 *
		2	143	24.20
		3	258	43.65
		4	120	20.30
		5	52	8.80

Note: * Class proportion <5% of the total sample (N < 30); ^†^ = equal variances and zero covariances; ^‡^ = equal variances and equal covariances.

**Table 4:** Descriptive statistics for the three classes identified through latent profile analysis.

Variable	Class 1	Class 2	Class 3
N	210	254	127
Demographics			
Gender (Male/Female)	104/106	150/104	104/23
Age (Years)	27.60 ± 9.74	26.03 ± 6.35	53.35 ± 8.99
Body Mass Index (kg/m^2^)	23.72 ± 4.12	23.37 ± 3.29	26.56 ± 4.43
Regular Physical Activity (Years)	13.53 ± 8.48	12.70 ± 7.26	29.53 ± 14.96
SF-36			
Social Functioning	48.51 ± 18.10	68.11 ± 22.76	82.68 ± 21.37
Mental Health	49.35 ± 13.82	70.54 ± 10.99	74.99 ± 13.00
Vitality	43.76 ± 13.49	60.04 ± 11.69	66.57 ± 12.29
Role Limitation—Physical	66.43 ± 35.41	80.71 ± 31.08	87.60 ± 24.78
Role Limitation—Emotional	42.06 ± 38.90	89.96 ± 20.79	92.39 ± 18.87
General Health	59.26 ± 16.21	71.52 ± 13.33	68.82 ± 10.59
Bodily Pain	76.05 ± 22.93	87.36 ± 15.04	84.82 ± 18.51
Physical Functioning	90.71 ± 14.33	97.80 ± 4.36	94.57 ± 8.45
GHQ-12			
Total Score	19.66 ± 4.32	14.85 ± 3.75	15.09 ± 3.90
Social Dysfunction	9.91 ± 2.66	7.67 ± 2.26	7.70 ± 2.35
Anxiety and Depression	7.21 ± 2.29	4.91 ± 2.14	5.01 ± 2.14
Loss of Confidence	2.54 ± 1.05	2.27 ± 1.01	2.39 ± 1.01
COPE-NVI-25			
Avoidance	13.67 ± 3.97	10.38 ± 3.52	9.46 ± 3.42
Positive Attitude	23.44 ± 5.92	26.97 ± 5.13	27.07 ± 6.40
Problem Orientation	18.08 ± 4.77	20.00 ± 4.04	21.35 ± 4.75
Transcendent Orientation	6.23 ± 4.29	6.09 ± 4.26	7.46 ± 5.23
Social Support	18.17 ± 6.49	20.06 ± 5.71	16.97 ± 6.22
AAQ-II			
Total Score	40.15 ± 8.33	26.33 ± 6.91	23.43 ± 6.93
I-CFQ			
Total Score	29.40 ± 7.87	17.65 ± 6.36	13.13 ± 5.41

Abbreviations: SF-36, 36-Item Short Form Survey; COPE-NVI-25, 25-Item Coping Orientation to Problems Experienced–New Italian version; GHQ-12, 12-Item General Health Questionnaire; AAQ-II, Acceptance and Action Questionnaire–II; I-CFQ, Italian Cognitive Fusion Questionnaire.

## Data Availability

The raw data supporting the conclusions of this article will be made available by the authors upon request. We chose to make the data available on request because the dataset contains individual-level health and psychological information collected from volunteer participants. Participants gave their consent for the use of their data for scientific and research purposes, and the study was approved by the Ethics Committee of Sigmund Freud University. We therefore preferred not to deposit the raw data in a public repository. They are available from the corresponding author upon reasonable request, in line with the original consent and the ethical approval.

## Supplementary Materials

The following supporting information can be downloaded at: https://www.mdpi.com/article/10.3390/sports14100452/s1. Figure S1: Standardized profiles for the three-profile sensitivity analysis excluding age, BMI, and years of regular physical activity; Figure S2: Standardized profiles for the three-profile sensitivity analysis excluding the SF-36 Social Functioning indicator; Table S1: (a) Fit index values for the sensitivity analysis excluding age, BMI, and years of regular physical activity. (b) Class sizes for the three-profile solution (retained).; Table S2: (a) Fit index values for the sensitivity analysis excluding the SF-36 Social Functioning indicator. (b) Class sizes for the three-profile solution (retained); Table S3: Fit index values for the alternative latent profile model specifications.
