# Supplementary material for: A Latent Profile Analysis of Coping, Psychological Inflexibility, and Health-Related Quality of Life in Adult Regular Exercisers
Source: Sports (Basel). 2026 Oct 13;14(10):452. doi: 10.3390/sports14100452 (PMC13709841; doi:10.3390/sports14100452)
Supplement: Supplementary file 1 [file sports-14-00452-s001.zip › sports-4467946-supplementary.pdf]

Table S1. (a) Fit index values for the sensitivity analysis excluding age, BMI, and years of regular physical activity. **(b)** Class sizes for the three-profile solution (retained).

(a)

Classes	AIC	AWE	BIC	CAIC	CLC	KIC	Entropy
1	75538.22	76031.71	75695.96	75731.96	75468.22	75577.22	1.000000
2	73883.36	74638.61	74124.36	74179.36	73775.11	73941.36	0.875551
**3**	**73373.06**	**74389.83**	**73697.32**	**73771.32**	**73226.80**	**73450.06**	**0.868257**
4	73204.06	74482.30	73611.57	73704.57	73019.85	73300.06	0.890598
5	72859.56	74399.28	73350.32	73462.32	72637.36	72974.56	0.899537

(b)

Class	N	%
1	90	15.23
2	286	48.39
3	215	36.38

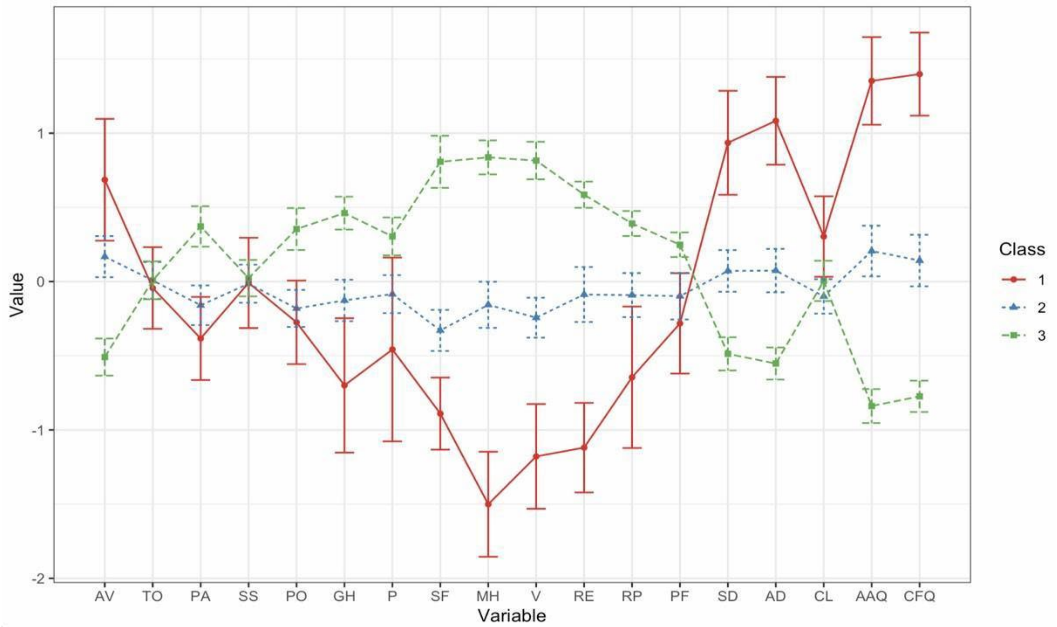

Figure S1. Standardized profiles for the three-profile sensitivity analysis excluding age, BMI, and years of regular physical activity.

Table S2. (a) Fit index values for the sensitivity analysis excluding the SF-36 Social Functioning indicator. **(b)** Class sizes for the three-profile solution (retained).

(a)

Classes	AIC	AWE	BIC	CAIC	CLC	KIC	Entropy
1	82790.20	83338.74	82965.47	83005.47	82712.20	82833.20	1.000000
2	81242.01	82079.86	81509.30	81570.30	81121.74	81306.01	0.864579
**3**	**80792.55**	**81919.39**	**81151.86**	**81233.86**	**80630.32**	**80877.55**	**0.886101**
4	80501.55	81917.46	80952.88	81055.88	80297.30	80607.55	0.872911
5	80284.18	81989.07	80827.52	80951.52	80037.97	80411.18	0.899536

(b)

Class	N	%
1	202	34.18
2	260	43.99
3	129	21.83

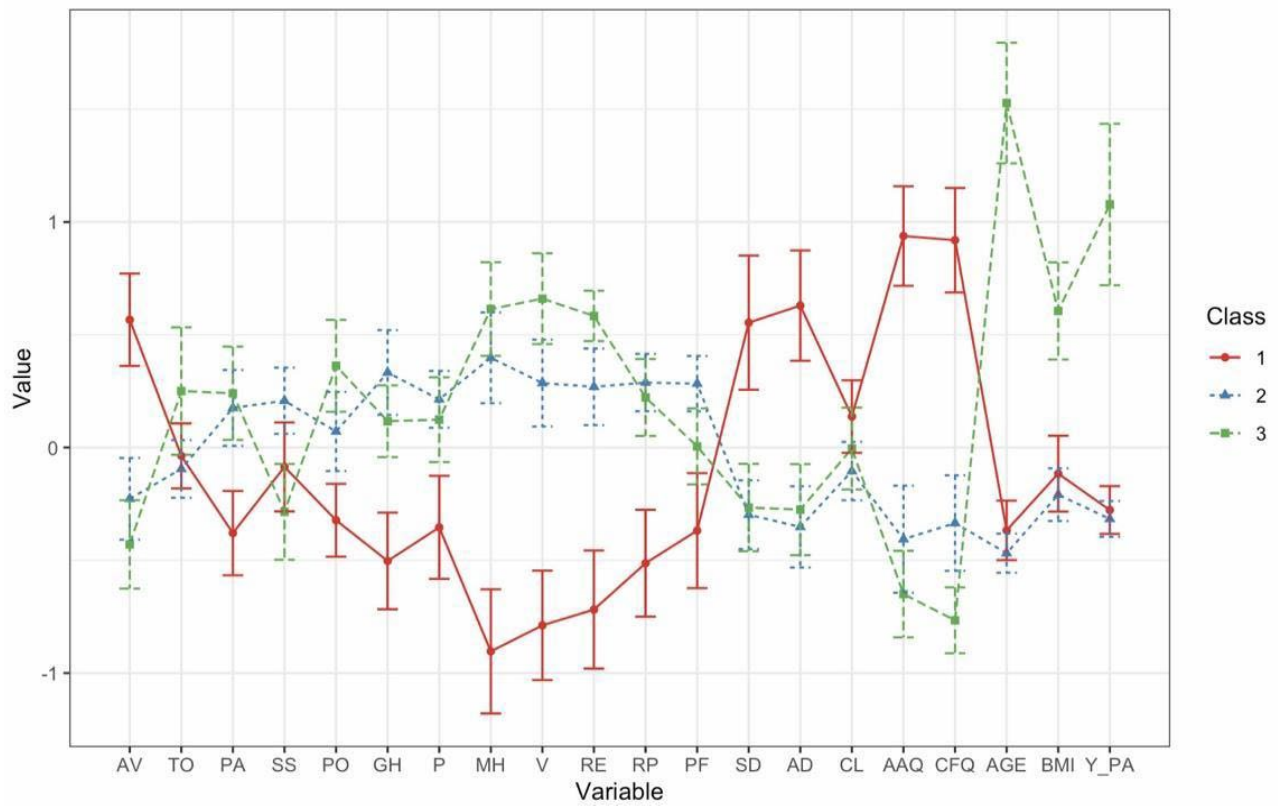

Figure S2. Standardized profiles for the three-profile sensitivity analysis excluding the SF-36 Social Functioning indicator.

Table S3. Fit index values for the alternative latent profile model specifications.

Model	Classes	AIC	AWE	BIC	CAIC	CLC	KIC	Entropy
1	1	88255.04	88831.11	88439.07	88481.07	88173.04	88300.04	1.000000
1	2	86533.45	87412.57	86813.89	86877.89	86407.20	86600.45	0.875938
1	3	86102.82	87284.75	86479.65	86565.65	85932.56	86191.82	0.869419
1	4	85682.23	87166.93	86155.46	86263.46	85467.99	85793.23	0.884741
1	5	85482.00	87269.47	86051.64	86181.64	85223.80	85615.00	0.901683
2	1	88254.54	88830.61	88438.57	88480.57	88172.54	88299.54	1.000000
2	2	–	–	–	–	–	–	–
2	3	–	–	–	–	–	–	–
2	4	–	–	–	–	–	–	–
2	5	–	–	–	–	–	–	–
3	1	84582.19	88048.62	85686.40	85938.40	84080.19	84837.19	1.000000
3	2	84590.43	88360.39	85791.04	86065.04	84043.69	84867.43	0.633712
3	3	83948.50	88020.62	85245.52	85541.52	83358.42	84247.50	0.960432
3	4	83948.52	88323.62	85341.94	85659.94	83314.26	84269.52	0.869668
3	5	83736.30	88414.28	85226.12	85566.12	83057.95	84079.30	0.826547
6	–	–	–	–	–	–	–	–

Note: Model 1 = equal variances and zero covariances; Model 2 = varying variances and zero covariances; Model 3 = equal variances and equal covariances. Model 6, allowing both variances and covariances to vary freely across profiles, could not be estimated within the Mclust framework implemented in tidyLPA. AIC, Akaike Information Criterion; AWE, Appropriate Weight of Evidence Criterion; BIC, Bayesian Information Criterion; CAIC, Consistent Akaike Information Criterion; CLC, Classification Likelihood Criterion; KIC, Kullback Information Criterion.
